# *LawVerse*: The Legal Framework for Clinical Metaverse Content

**DOI:** 10.1089/jmedxr.2024.0028

**Published:** 2025-01-24

**Authors:** Barbara Pasa, Alessandro Bernes, Andrea Gaggioli, Emanuele Tuccari, Fabiana Zollo, Giorgia Vulpiani, Loris Pignolo, Antonio Scala, Antonio Cerasa

**Affiliations:** 1 Università IUAV di Venezia, Venice, Italy.; 2 Ca’ Foscari University of Venice, Venice, Italy.; 3 Research Center in Communication Psychology, Catholic University of Milan, Milan, Italy.; 4 Applied Technology for Neuro-Psychology Lab, IRCCS Istituto Auxologico Italiano, Milan, Italy.; 5 University of Pavia, Pavia, Italy.; 6 University of Macerata, Macerata, Italy.; 7 S. Anna Institute, Crotone, Italy.; 8 CNR-ISC, Applico Lab, Rome, Italy.; 9 Big Data in Health Society, Rome, Italy.; 10 Institute of BioImaging and Complex Biological Systems (IBSBC-CNR), Catanzaro, Italy.

**Keywords:** metaverse, legal issues, data protection, intellectual property rights, non-fungible tokens, medical device

## Abstract

The term *MedVerse* has been coined to describe how the infrastructure layers of the metaverse can be adopted in the health care sector, to enable the distribution of contents and interact with patients. In the last 2 years, a large amount of proof-of-concept metaverse-based technologies and applications have been proposed in several medical realms. However, the advent of such an expensive, hypercomplex technology promoted as a new instrument with a strong incentive to invest time and money in digital items (driving revenue for some categories) poses several legal challenges. Here, we discuss the main legal issues (the so-called *LawVerse*) related to their application to the clinical metaverses realms.

## From Metaverses to the *MedVerse*

The European Union (EU) Parliament^1^ defines metaverses as “*digital simulations of multidimensional spaces that can be based on visual, auditory, and tactile perception. They can simulate digitised reality, mirror worlds, digital twins, or be entirely decoupled from the physical layer and populated with AI algorithms*.” The metaverse has garnered significant attention from the research community lately, and a substantial amount of work has already been done on its ideas, design, and applications in several domains. In the last 2 years, there have been numerous attempts to develop new syllogisms with the goal of more clearly illustrating how this communication technology might be used. What emerged from this preliminary evidence was that moving from a *Psycho-Verse*^2^ to a *Meta-Health*^3^ or *Med-Verse*^4^ perspective, the health care sector will be one of the main domains exploiting this kind of technology.^5^

The metaverse is a “federation” of multiple technologies (artificial intelligence [AI], tangible and multimodal interfaces, blockchain, and Internet of Things) shared by multiusers simultaneously to connect the 3D environments to cyber-physical devices and their data, thus enabling seamless interaction between the “virtual” and “real” world (also called, *phygital space*^6^) managed by AI algorithms. The two-way connection between the virtual and real worlds is provided by the digital twins: digital copies of real-world objects, systems, or processes that are in sync with the physical world. This implies that experiences in the virtual world might influence physical actions and vice versa. Furthermore, any changes made to the real world are mirrored in their virtual counterparts, enabling reciprocal feedback. For example, engaging with an avatar in the virtual world can cause haptic feedback to occur in the real world.^4^

The utilization of the metaverse in clinical medicine will be a novel approach to methods of treatment, providing some benefits concerning previous approaches (such as telemedicine or traditional individual virtual reality (VR) sessions).^7^ For instance, while telemedicine provides routine treatment with convenience and accessibility, the metaverse transforms how patients and doctors engage with medical information. Depending on the clinical setting, telemedicine is a good option for routine care, while metaverse tools are best suited for complex simulations and immersive social training.^8^ Another advantage is the ability to construct controlled, multiuser, and customized therapeutic environments that can be adapted to the unique requirements and conditions of each patient. Using AI tools, the metaverse also could enable the tailoring of therapeutic situations and surroundings to each patient’s unique requirements, preferences, and treatment objectives. In mental health care, for instance, where the subjective nature of diseases requires individualized approaches for best results, this customization is essential. For instance, in the neurorehabilitation domain, the advent of the metaverse will allow to start a new era of treatments moving from standard individual therapy to multiusers therapeutic sessions controlled in a shared virtual world.^9^ In the psychiatric domain, the employment of this “*service-oriented architecture that emphasizes social and content aspects will be used to exploit the ‘synchronized brains’ potential exacerbated by social interactions*.”^10^ However, before translating this “federation” of multiple technologies into the medical realm, we need to handle legal matters using a risk-adjusted strategy, keeping in mind that there can be exceptions to accommodate medical concerns (such as privacy, data protection, financial flexibility, decentralization, security, and intellectual property rights) as this technology is still in its infancy.

## From the *MedVerse* to the *LawVerse* Framework

Metaverses will involve an extension of the legal and ethical issues that have arisen in research on virtual health communities, telehealth, and AI in health care.^11,12^ Issues are not only connected to privacy and data protection but also to safe technological development and production, as well as a correct medical diagnosis and liabilities in contract and tort, due to the instrumental and supporting role played by new digital tools in the doctor–patient relationship, by considering the higher risks to which individuals might be exposed.

Furthermore, metaverses—as immersive and constant virtual 3D worlds, where people are actively involved in the creation of virtual worlds and interact by means of digital humans or digital twins of persons to carry out a wide range of activities—open many opportunities as well as challenges in other legal areas, such as intellectual property laws. The US Congress^13^ and the European Parliament^1^ both showed interest in legal matters brought up in the setting of metaverses. They raised apprehensions about the potential of metaverses to replicate and exacerbate existing challenges associated with contemporary online service platforms and applications. These concerns encompass issues such as the presence and moderation of illicit and harmful content online, the responsibility for consumer manipulation through advertising practices, data protection and privacy concerns, competition, and the protection of intellectual property rights. Additionally, there are concerns related to the ownership or contractually granted control of digital assets within the metaverses, legal complexities surrounding smart contracts, non-fungible tokens (NFTs), and the use of digital currencies involving virtual money transfers between avatars. Further worries extend to areas such as money laundering and gambling, as well as security issues arising from potential connections between the dark web and the metaverses.

**FIG. 1.**
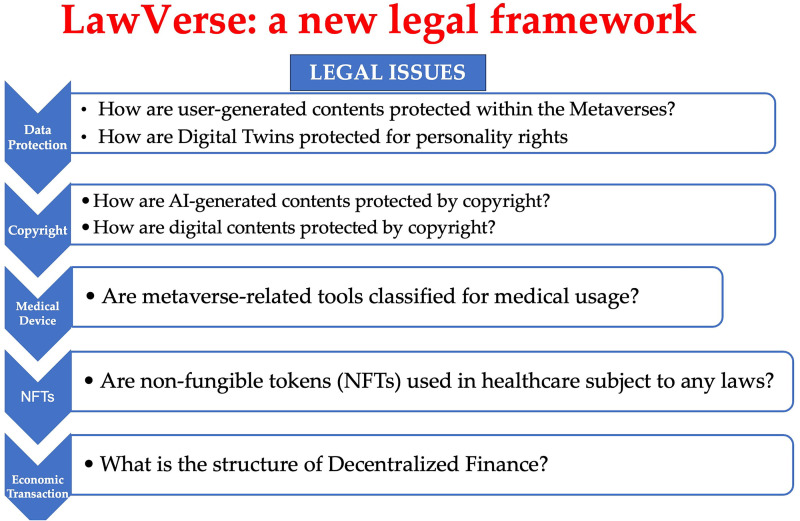
Summary of the main legal issues related to the application of metaverse technologies in health care. Framework of the LawVerse layers.

For all these reasons, in this commentary, we described some legal concerns that underpin the use of the so-called *LawVerse* in public services, especially in the health care sector and medical setting (Fig. 1).

### Data protection

Addressing the storage, handling, and protection of data in the metaverse is crucial, accompanied by accountability for potential data theft or misuse. In this regard, regulations and proposals are flourishing not only at supranational level—such as the General Data Protection Regulation (“GDPR”),^14^ the “Artificial Intelligence Act” (“AI Act”),^15^ and a Regulation on the “European Health Data Space”^16^—but also at national level, with the presentation of a series of interesting legislative proposals in the different EU Members States. Compliance with the “GDPR”—in case of special categories of personal data—necessitates “*explicit user consent for each specific purpose*,” varying based on activities within the metaverse (Art. 9). Concerns arise as users’ data are expected to be collected more extensively and continuously during their metaverse experiences, potentially making involuntary and ongoing consent challenging.

Furthermore, according to Art. 9, par. 4, “GDPR,”^14^ “M*ember States may maintain or introduce further conditions, including limitations, with regard to the processing of genetic data, biometric data or data concerning health*.” Therefore, data security measures—to protect information security and individual rights in the new environments—must also play a central role (at national and supranational levels).^17^

Additionally, defining the roles of data controllers and processors in the metaverse poses a significant challenge due to the intricate interconnections among entities in each virtual universe. Establishing clear distinctions regarding responsibilities and actions on behalf of users becomes a complex task in this interconnected environment. The decentralized approach, where users have control over their data and its sharing, may offer potential solutions to data protection issues that are intricate in more centralized business models.^18^

The European “AI Act”^15^ appears to be focused on a “risk-based approach”: there is an absolute ban on placing certain AI systems on the market or putting them into service or use (see Art. 5, “Prohibited AI practices”), but there are also specific obligations provided for AI systems “admitted,” but classifiable as “high risk.” According to Art. 6 para 1, an AI system that meets both of the following conditions is classified as high risk: *“a) the AI system is intended to be used as a safety component of a product, or the AI system is itself a product, covered by the Union harmonization legislation listed in Annex I; b) the product whose safety component pursuant to point (a) is the AI system, or the AI system itself as a product, is required to undergo a third-party conformity assessment, with a view to the placing on the market or the putting into service of that product pursuant to the Union harmonization legislation listed in Annex I*.” With regard to the first condition, Annex I explicitly includes the EU Medical Device Regulation 2017/745 (“MDR”)^19^ among the harmonizing regulations. Regarding the second condition, under the “MDR” all “high-risk” devices must undergo conformity assessment by a third party (Notified Body) in order to be placed on the market.

Therefore, a medical device that incorporates or is itself an AI system will be considered a “high-risk” AI system. In addition to the high-risk AI systems referred to in Art. 6, para 1, the AI systems listed in Annex III (*inter alia*, in the “biometrics” area) are “high risk” (Art. 6, para 2). However, according to Art. 6, para 3, “*an AI system referred to in Annex III shall not be considered to be high-risk where it does not pose a significant risk of harm to the health, safety or fundamental rights of natural persons, including by not materially influencing the outcome of decision making*.”^19^ Thus, in line with the “risk-based approach” mentioned above, high-risk AI systems will be allowed on the European market subject to compliance with certain mandatory requirements (such as management system and transparency obligations toward users) and a preliminary conformity assessment. This assessment of software compliance may raise many other data protection issues (e.g., in relation to deidentification techniques used, security measures taken; see Article 10, para 5, EU AI Act).^15^ Metaverses (and in particular “MedVerse”)—as a “federation” of multiple technologies (AI, tangible and multimodal interfaces, blockchain, and Internet of Things)—could be considered a “high-risk” environment.^20^

### Personality rights and Intellectual Property rights

The digital twin of a person (DToP) not only replicates a distinctive person but also constitutes a nearly instantaneous synchronized multipresence.^21^ This entails the capability to exist simultaneously in various locations within both the digital and physical realms. The DToP generates an intricate virtual model mirroring the physical person. This is achieved through sensors that transmit information or two-way Internet of Things connections, enabling synchronization between the digital and physical environments. It receives real-time updates and employs simulation, machine learning, and reasoning to support decision-making processes. Any alterations in the tangible world are mirrored in the digital representation of the twin. Due to these factors, DToP holds significant disruptive potential in the medical field. Additionally, through decentralized and encrypted databases, DToP technologies enable secure storage and transmission of data, ensuring that only the data owner can make any alterations. These technologies are integral to the metaverse concept, serving as a means for decentralized recording of digital ownership.

The issue of recognizing intellectual property rights for “works” generated by AI systems raises broader questions about the structure of copyright. This prompts a comprehensive evaluation, focusing on two fundamental queries: whether the input/output of the algorithmic processes can be legally appropriated and recognized by copyright, and how the notions of “free”/protected expressions of ideas should be understood in the era of generative AI.

### Medical device: Metaverse-related applications certified for medical purpose

When the use of an electronic medical device takes place in the context of medical treatment, it usually seems to serve as a tool that helps to make decisions that pertain to the health professional. However, the application of certain regulations will differ depending on the purpose of each specific tool. The versatility of metaverse-enabled technologies raises the question of whether certain associated tools should even be classified as means for medical use. Depending on the factual circumstances, for example, an augmented reality (AR) headset worn by the patient may or may not meet the criteria of a medical device or its accessory. Only if the destination of the device meets some requirements it should be subject to medical device certification and all the safety and quality requirements set out by the European Regulation.^19^ On the contrary, if the software acts just as an advanced videoconference tool, it would be difficult to consider them a medical device, considering the sole communication purpose between physicians and patients.

The European AI Act^15^ represents an ambitious attempt to regulate the risks arising from the structural and proven opacity, complexity, data dependency, and capacity for autonomous decision-making behavior of AI systems. In the perspective chosen by the European legislator, however, the space devoted by the regulation to the protection of the person appears rather meagre, at least if one looks at the risk that the technologies related to eHealth may turn into a means through which the therapeutic alliance, which is an immediate corollary of the care relationship, may be undermined or emptied of content. The question is whether the care relationship continues to be structured exclusively on the doctor–patient pair or should the IA system also be included in the middle. Also, the U.S. Food and Drug Administration (FDA), in addressing the challenges related to Software as^20^ a Medical device have considered that AI should not replace physician decision-making, but be used to assist clinicians. Future legal research should address how to identify the conditions for comprehensible but at the same time comprehensive information of AI, as well as a proportionate, reasonable, and appropriate use of intelligent medical devices, which does not renounce certain aspects of our humanity, in a new “digital humanism,” for a medicine “with” machines and not “of” machines.

Another legal question arises from the possible serious harm to which the patient is exposed because of the concrete usage of digital devices within the treatment. Who is liable for damage caused to the patient by the incorrect evaluation of the AI medical device used? The European AI Act^15,16^ (Art. 6, par. 2,) affirmed that the use of such AI systems should be classified as high risk since they are intended to be used as a safety component of a product, or the AI system is itself a product that requires a third-party conformity assessment pursuant to the Union harmonization legislation (EU Regulation 2017/745).

### NFTs, intellectual property, and liability issues

We must also consider the legal issues connected to those digital assets and products that are defined as “non-fungible”: the NFTs. There is no specific legislation on these tokens, so many legal issues related to this type of asset arise and they primarily concern identifying their juridical nature. U.S. legal doctrine qualifies NFTs as digital personal properties, affirming the need to treat NFTs as items of actual personal property, with the subsequent applicability of the regulation of the sale of personal property, in such a way as to clearly distinguish the legal situation relating to NFTs from that relating to licenses on intellectual property.^22^ In fact, according to this theory, property regulation is better suited to how NFTs are used, as the owner can enjoy and dispose them without any external interference. This would conflict with the online intellectual property license model, where the owner of a work’s intellectual property rights can decide how the copyright can be used or sold.^23^ This approach is shared, for example, by the High Court of the United Kingdom which ruled that NFTs “*are to be treated as property as a matter of English law*” (case *Osbourne v. Persons Unknown and Ozone Inc.*).^24^ According to a different theory, NFTs could be classified as atypical debt securities, attributable to the documents of legitimation used to identify the person entitled to the service since the contract is formed in a separate act.^25^ According to this reconstruction, an NFT does not incorporate the digital content transferred between the parties. Still, it represents only a computer sequence subjected to a hashing process and some algorithmic properties of the token. This certificate is then uniquely connected via a link to an off-chain site where the digital product, an object of the transaction, is stored. Additionally, the smart contract is limited to executing the contractual provisions governed by the parties in separate natural language contracts. Therefore, the NFT would not incorporate any rights but would result in an enabling title allowing access to digital content. This approach leads the NFT to a sort of digital key that allows access, for instance, to the “*hotel room booked on the basis of a natural language contract with the manager of the accommodation facility*,” allowing those identified as entitled to benefit from the digital content.^26,27^

### Economic transactions: The decentralization opportunity

The metaverse is characterized by social life and technological exchange, mainly regulated by blockchain technologies. Blockchain is a decentralized distributed ledger technology that stores transactional data across multiple computers in an unchangeable manner. A blockchain is a chain of safe data composed of discrete blocks, each of which comprises a time stamp, a list of transactions, and a cryptographic hash of the block before it. By accomplishing this, data security, integrity, transparency, and interoperability across various health care systems are ensured, making it resistant to manipulation and fraud.^28^

Activities in metaverses are not controlled by a single company or provider. These digital worlds are governed by decentralized autonomous organizations (DAOs), which are organizations without hierarchy or a chief executive officer (CEO).^29^ Decisions are collectively made by their members, who each have a stake in the organization. The tool enables individuals to collaborate and organize with strangers globally, promoting transparency and democratic management of spaces through specific consensus mechanisms.^29^ These mechanisms are designed to prevent any individual from changing or manipulating anything without the group’s approval. DAOs eliminate the need to trust individuals within the group. Instead, trust is placed solely in the DAO’s code, which is completely transparent and verifiable by anyone.

## Conclusions

The evolving nature of metaverses introduces a multifaceted legal environment that necessitates comprehensive frameworks and perhaps new practices to address intellectual property rights, personality rights, product safety, data protection, contract and tort liabilities, and operational resilience concerns. All concerns have a supranational or international scope and go beyond the perspective of a particular legal system. Examples of such challenges include how to create policies from a comparative standpoint and what laws should be in place to sufficiently safeguard intellectual property rights and privacy in metaverses. An adequate (supranational) legislation is undoubtedly one step away from the first European debate. The competition among the world’s legal systems (e.g., the FDA) will undoubtedly enhance the way this technology is managed in the future.

## Abbreviations Used

AR: augmented reality
CEO: chief executive officer
EU: European Union
VR: virtual reality
